# Supplementary material for: The causal role of circulating inflammatory markers in osteoporosis: a bidirectional Mendelian randomized study
Source: Front Immunol. 2024 Jul 18;15:1412298. doi: 10.3389/fimmu.2024.1412298 (PMC11291241; doi:10.3389/fimmu.2024.1412298)
Supplement: Supplementary file 1 [file DataSheet_1.zip › supplementary material/OSTEOPOROSIS-CIM/GCST90274791.tsv.gz_leave_one_out.pdf]

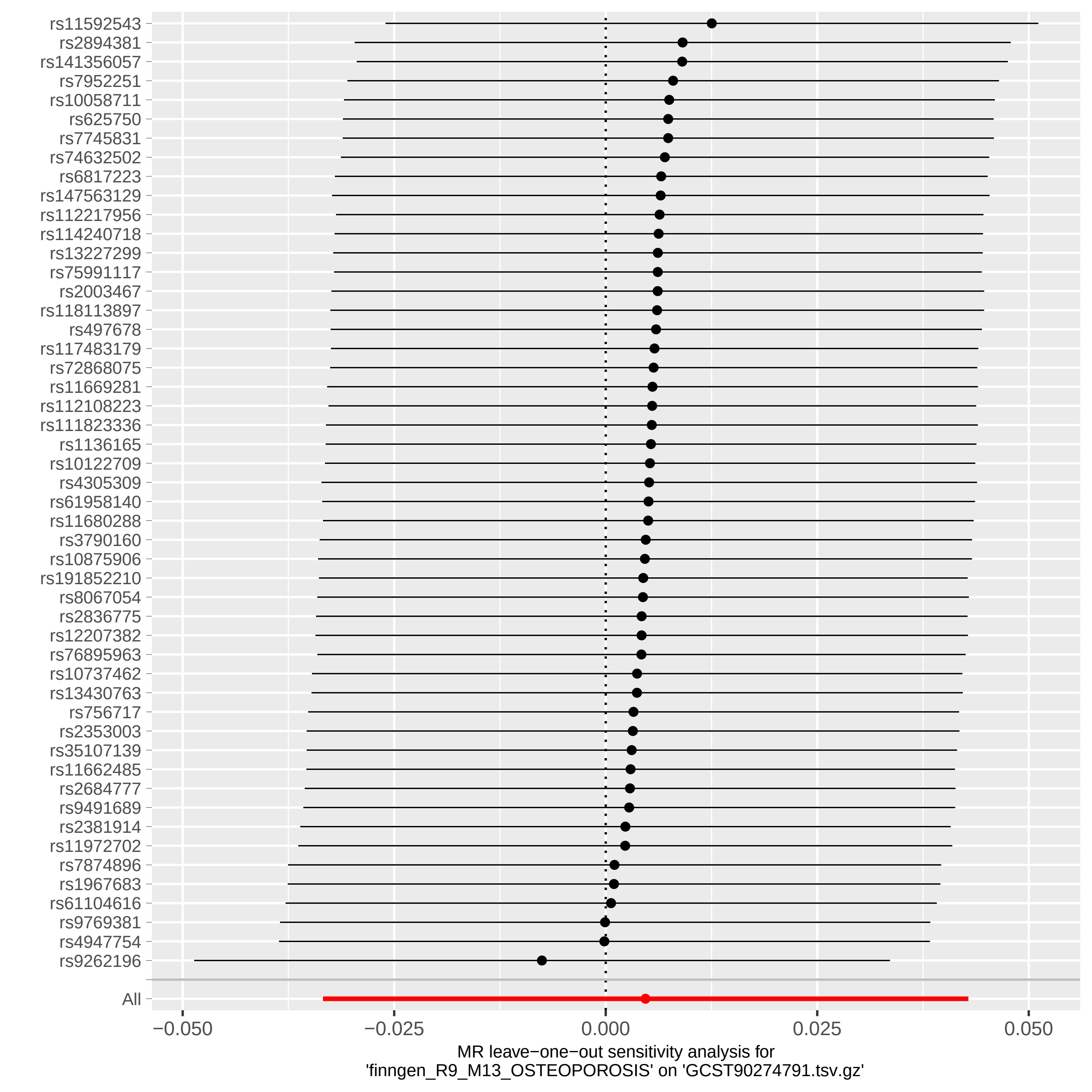
rs11592543
rs2894381
rs141356057
rs7952251
rs10058711
rs625750
rs7745831
rs74632502
rs6817223
rs147563129
rs112217956
rs114240718
rs13227299
rs75991117
rs2003467
rs118113897
rs497678
rs117483179
rs72868075
rs11669281
rs112108223
rs111823336
rs1136165
rs10122709
rs4305309
rs61958140
rs11680288
rs3790160
rs10875906
rs191852210
rs8067054
rs2836775
rs12207382
rs76895963
rs10737462
rs13430763
rs756717
rs2353003
rs35107139
rs11662485
rs2684777
rs9491689
rs2381914
rs11972702
rs7874896
rs1967683
rs61104616
rs9769381
rs4947754
rs9262196
All
−0.050
−0.025
0.000
0.025
0.050
MR leave−one−out sensitivity analysis for
'finngen_R9_M13_OSTEOPOROSIS' on 'GCST90274791.tsv.gz'
